# Media Exposure, Disaster Experience, and Risk Perception of Rural Households in Earthquake-Stricken Areas: Evidence from Rural China

**DOI:** 10.3390/ijerph17093246

**Published:** 2020-05-06

**Authors:** Dingde Xu, Linmei Zhuang, Xin Deng, Cheng Qing, Zhuolin Yong

**Affiliations:** 1 Sichuan Center for Rural Development Research, College of Management of Sichuan Agricultural University, Chengdu 611130, China; 2 College of Management of Sichuan Agricultural University, Chengdu 611130, China; zhuanglinmei@stu.sicau.edu.cn (L.Z.); qingchen@stu.sicau.edu.cn (C.Q.); zhuolinyong@stu.sicau.edu.cn (Z.Y.); 3 College of Economics of Sichuan Agricultural University, Chengdu 611130, China; dengxin@sicau.edu.cn

**Keywords:** media exposure, disaster experience, risk perception, earthquake, Sichuan province, rural China

## Abstract

For effective communication and management of disaster risks, it is important to explore how media exposure and disaster experience related to earthquake events affect residents’ prospect ranks of disaster risk perceptions. Using survey data from 327 households located in the Wenchuan and Lushan earthquake regions in China, the ordinary least square method was used to explore the associations among media exposure, severity of disaster experience, and residents’ perception of prospect ranks of the possibility and severity of disasters. The results showed the following. (1) Rural households relied predominately on television broadcasts from traditional media, and on mobile phones and internet content from new media to obtain disaster information. From the residents surveyed, 90% believed that a disaster experience was serious, 82% considered that another major earthquake would seriously affect their lives and property, while approximately 40% of the residents did not believe there would be another major earthquake in the next 10 years. (2) Media exposure was negatively correlated with the perceived prospect ranks of the probability and severity of disasters, with traditional media exposure significantly negatively correlated with the perceived prospect ranks of the severity of disasters and new media exposure significantly negatively correlated with the perceived prospect ranks of the probability of disasters. Severity experience was significantly and positively correlated with the perceived prospect ranks of the probability and severity of disasters. (3) New media exposure moderated the relationship between residents’ disaster experience and their perception of prospect ranks of the severity of disasters. This study can help deepen our understanding of disaster risk communication and better guide the practice of disaster risk management.

## 1. Introduction

Faced with the threat of natural disasters, many empirical studies have shown that effective and adequate disaster preparedness plays an important role in preventing the loss of life and damage to property [1,2,3,4,5]. For example, Godschalk et al. [6] found that a $1 investment in disaster relief can yield a $4 gain. However, studies have found that residents in vulnerable communities (communities with limited resources and which are often threatened and impacted by earthquakes and their secondary disasters) are not adequately prepared for external disasters (e.g., [7,8,9]). One important reason for this is the lack of effective communication of information to people who perceive the disaster risk to be of a low level. Media exposure is one of the most essential methods for residents to obtain disaster information [7,10]. With an increasing occurrence of global disasters, it is important to understand the types of media platforms that residents generally use to obtain disaster information and how frequently these platforms are accessed [11,12], especially for vulnerable rural households in developing countries.

The perception of disaster risk is an important driving factor to consider for understanding the behavioral decisions that residents make in disaster-prone areas. Therefore, research on the factors influencing disaster risk perception has become an essential topic in the disaster risk management field [13,14,15]. Many studies have focused on the impact of individual (e.g., [16,17,18]), family socio-economic (e.g., [19,20]) and village characteristics (e.g., [21,22]) on residents’ disaster risk perceptions. Because it is an important factor that affects residents’ risk perceptions, disaster information is often incorporated into models as a control variable (e.g., [23,24]). However, few empirical studies have specifically focused on the impact that media exposure has on residents’ risk perceptions of disasters [10,12] and few studies have explored the associations among traditional and new media exposure and residents’ disaster risk perceptions [10]. Current studies take media exposure and residents’ disaster experience as indicators that directly affect residents’ disaster risk perceptions. However, few empirical studies have confirmed that there is a moderating effect between media exposure and disaster experience that indirectly affects residents’ disaster risk perception [10]. In China’s vast earthquake disaster threat areas, further research is required to understand the moderating effect between media exposure and disaster experience, and how these affect residents’ disaster risk perception.

**Figure 1:**
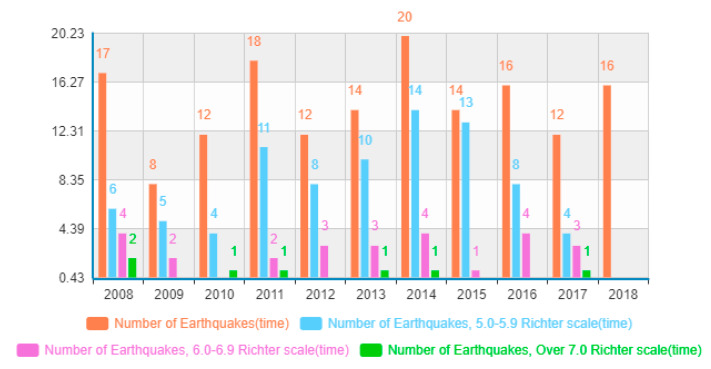
Statistics on the number of earthquakes in China from 2008 to 2018.

China is a large country with mountainous areas, where a large number of people live [25,26,27]. Affected by geological movement, China has suffered frequent earthquake disasters in recent years, causing a large number of deaths, serious damage to property, and far-reaching impacts on social and economic development. In the past 10 years, the country has experienced 159 earthquakes of at least magnitude 5 on the Richter scale. Among these, there have been seven major earthquakes with magnitudes above 7, 26 strong earthquakes with magnitudes between 6 and 6.9, and 83 moderate earthquakes with magnitudes between 5 and 5.9 (Figure 1). These earthquake disasters resulted in more than 48,000 casualties and 1.13 trillion Yuan in economic losses. The Wenchuan and Lushan earthquakes were the deadliest among the seven major earthquakes. For instance, the Wenchuan earthquake in 2008 caused about 450,000 casualties and direct economic losses as high as 845.2 billion Yuan [28]. The disaster risk management of China’s Sichuan earthquake disaster area was relatively small; therefore, relevant research is urgently required [24,29].

The present study focused on the areas most damaged by the Wenchuan and Lushan earthquakes in Sichuan province, China. The first main objective was to analyze the media exposure, disaster experience and disaster risk perception characteristics of residents in the worst-hit earthquake areas. The second objective was to build an econometric model to explore the correlation among media exposure, disaster experience, and prospect ranks of disaster risk perception, and to further subdivide the types of media exposure (traditional and new media).

## 2. Theoretical Development

The purpose of this study is to explore the correlation between media exposure, residents’ disaster experience and residents’ prospect ranks of disaster risk perception in the earthquake disaster threat area. In this study, media exposure refers to the channels through which the residents of the earthquake disaster threat area obtain disaster information at different stages of the disaster, which can be specifically divided into new media exposure and old media exposure [10]. Among them, new media exposure refers to the disaster information obtained by residents mainly through new media channels (mobile phones and the Internet), while old media exposure refers to the disaster information obtained by residents mainly through traditional channels (TV, magazines, newspapers, radio). Residents’ disaster experience refers to the severity of the impact of the most severe earthquake disaster on their family in their memory [24,29]. Residents’ perception of disaster risk refers to their attitude towards disaster risk and their intuitive judgment of disaster risk [24,29]. In the follow-up part of this part, the study will systematically sort out the existing studies on the correlation between media exposure, residents’ disaster experience and residents’ prospect ranks of disaster risk perception, and put forward the research hypothesis of this study on this basis.

### 2.1. Media Exposure and Perceived Prospect Ranks of Disaster Risk Perception

In the face of disaster threats, media exposure is one of the most important ways for residents to judge and understand disaster information [10,12,30,31]. Media exposure has multiple functions in the formation of disaster risk perception, such as communicating information before, during, and after the disaster; encouraging residents to learn disaster knowledge and skills; and establishing public responsibility and safety culture [10,32,33,34]. The speed of accurately reporting and disseminating information by the media will affect residents’ perception of disaster risk, and thus affect their behavioral decisions [35,36]. Meanwhile, the rapid spread of disasters in a certain area will further convey social norms [10,32]; through communication among the public, a culture of responsibility and safety can be established to improve public awareness of disaster prevention and risk reduction [10,32,37,38]. In addition, people in disaster-prone regions will learn about disaster prevention and risk reduction knowledge, and how to prepare for disasters in advance [39,40,41].

**Figure 2:**
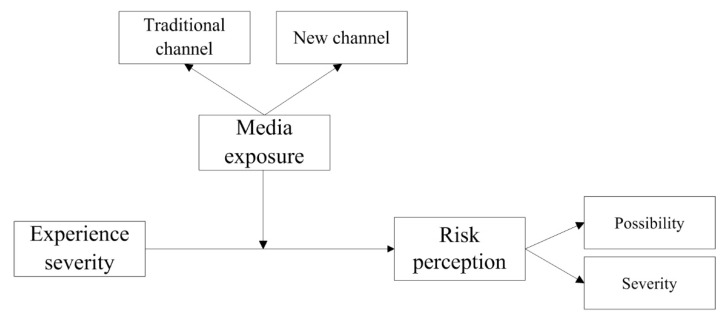
Theoretical framework of media exposure, disaster experience severity and risk perception.

From the analysis of information channels (such as Internet, TV, newspapers, radio, etc.,) to the formation process of residents’ disaster risk perception, it can be seen that residents’ disaster risk perception is closely related to the frequency of information received (including the speed of transmission) and the quality of information (information credibility and usefulness) [31,42]. Modern society is an information age society, where traditional media and new media coexist [36,43]. Due to the characteristics of fast transmission speed and low cost, new media has become increasingly important in the field of disaster risk communication [31,44], and, especially when residents think that the mainstream media cannot provide enough information, the use of new media plays a crucial role in the effective transmission and communication of disaster information [31,42,45]. In essence, channel residents choose to obtain information from or rely more on certain channels in order to accurately grasp the actual situation of disasters and reduce the uncertainty in the process of disaster information transmission [7,10]. From the existing studies, it is generally believed that media exposure can significantly improve the disaster risk perception of residents [10,34,46,47]; media exposure here includes both traditional media exposure and new media exposure. For example, Fleming et al. [46], Morton and Duck [47] found that traditional media exposure, such as to newspapers, has a positive significant effect on residents’ disaster risk perception; Zhu and Yao [34] found that new media exposure has a positive influence on residents’ disaster risk perception; Hong et al. [10] also found that the media exposure (including traditional and new media exposure) has a positive significant effect on the perceived severity of disasters. As such, this research proposes hypothesis 1 (H1, H1a, H1b) (Figure 2).

**H1:**  *Media exposure is significantly and positively correlated with the perceived prospect ranks of probability and severity of disasters.*

**H1a:**  *Traditional media exposure is significantly and positively correlated with the perceived prospect ranks of probability and severity of disasters.*

**H1b:**  *New media exposure is significantly and positively correlated with the perceived prospect ranks of probability and severity of disasters.*

### 2.2. Disaster Experience and Perceived Prospect Ranks of Disaster Risk Perception

Many empirical studies show that residents’ disaster risk perception will be affected by their direct or indirect disaster experience [10,22,48]. For people with rich disaster experience, when the disaster occurs again, theoretically, they can quickly judge the disaster situation based on their previous experience, that is, people with rich disaster experience generally have more rational disaster risk perception [10,22]. They will obtain disaster information through media and other channels and make reasonable decisions on disaster prevention and mitigation [10]. Therefore, in existing studies, most scholars believe that disaster experience is significantly and positively correlated with disaster risk perception (e.g., [12,48,49]). For example, Xu et al. [22] found that there was a positive correlation between residents’ landslide disaster experience and the perceived probability of disasters. Botzen et al. [50] found that there was a positive correlation between residents’ flood disaster experience and their risk perception and insurance needs. However, for people without disaster experience, they usually construct their disaster risk perception through disaster information obtained from the outside world, and then make decisions on disaster prevention and reduction. At this point, the external information may completely magnify their perception of disaster risk. Therefore, some studies have found that media information has an impact on residents’ disaster risk perception, but such impact is only significant in the group with low disaster experience [51]. Additionally, a few studies have found that the correlation between residents’ disaster experience and disaster risk perception is not significant (e.g., Xu et al. [22]). However, most studies believe that there is a significant positive correlation between the two. Based on this, this research proposes hypothesis 2 (H2) (Figure 2).

**H2:**  *Residents’ disaster experience is positively correlated with the perceived prospect ranks of probability and severity of disasters.*

### 2.3. Media Exposure Moderates the Relationship between Residents’ Disaster Experience and Perceived Prospect Ranks of Disaster Risk Perception

Most studies suggest a significant positive correlation between residents’ disaster experience and perceived prospect ranks of disaster risk perception. However, some studies suggest that this correlation is indirect. For example, faced with the threat of disasters, Hong et al. [10] believed that individuals with adequate disaster experience could identify disaster information more calmly and effectively, and respond with quicker behavioral decisions. The more pronounced the residents’ disaster experience was, the weaker the correlation between media exposure and perceived prospect ranks of disaster risk perception. Kasperson et al. [52] argued that individuals lacking disaster experience would rely more on the content of external information to magnify their perception of disaster risk (for example, they may think that disasters are more likely to occur and be more serious). Based on this, this research proposes hypothesis 3 (H3, H3a, H3b) (Figure 2).

**H3:**  *Media exposure moderates the relationship between residents’ disaster experience and their perceived prospect ranks of possibility and severity of disasters. When disaster experience is high, the positive relationship between media exposure and the perceived prospect ranks of possibility and severity of disasters are strong.*

**H3a:**  *Traditional media exposure moderates the relationship between residents’ disaster experience and their perceived prospect ranks of possibility and severity of disasters. When disaster experience is high, the positive relationship between traditional media exposure and the perceived prospect ranks of possibility and severity of disasters are strong.*

**H3b:**  *New media exposure moderates the relationship between residents’ disaster experience and their perceived prospect ranks of possibility and severity of disasters. When disaster experience is high, the positive relationship between new media exposure and the perceived prospect ranks of possibility and severity of disasters are strong.*

## 3. Material and Methods

### 3.1. Data Sources

**Figure 3:**
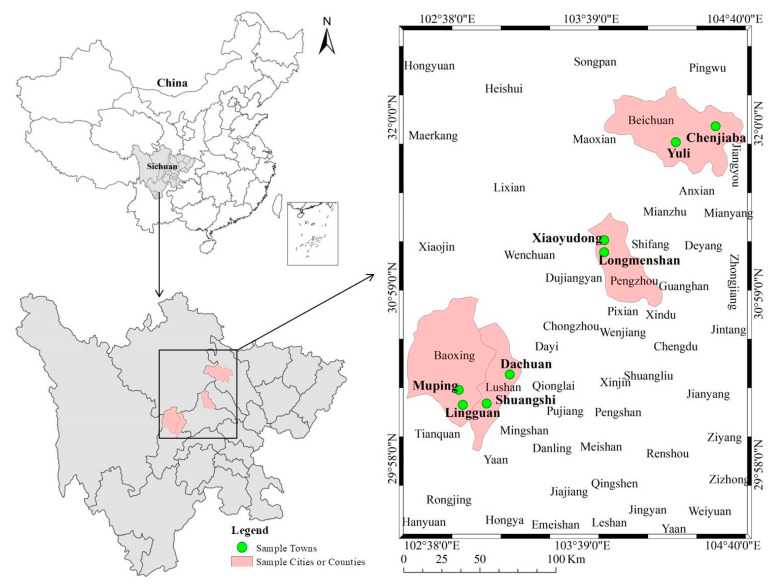
Location map of sample counties and towns (Figure source please see [53]).

The data used in this research were mainly acquired from a questionnaire survey conducted by the research group in areas affected by the Lushan and Wenchuan earthquakes in July 2019. The study used one-on-one interviews to evaluate behavioral responses to disaster risk perception and disaster preparedness. To ensure that representative and non-biased samples were tested, stratified random sampling was performed. A total of 327 households were investigated from 16 villages of eight townships in four counties. For a detailed introduction, please refer to Xu et al. [24]. See Figure 3 for the location of the sample counties and towns.

### 3.2. Measures

#### 3.2.1. Media Exposure

Media exposure mainly reflects public access to media information in the worst-hit earthquake areas. Referring to the studies by Fleming et al. [46], Hong et al. [10] and Lee [32], this research divided rural household media exposure into traditional and new media forms. Traditional media includes newspapers, magazines, radio, and television, while new media includes mobile phones and the internet. These variables were measured by asking residents how often they used these media platforms (Table 1). After obtaining this information, the indicators of traditional and new media exposure were summed and averaged, and the mean value was substituted for the scores of the two groups, as performed by Hong et al. [10]. At the same time, all indicators representing media exposure were summed and averaged, and their mean value was substituted for media exposure.

#### 3.2.2. Disaster Experiences

On the measurement of disaster experience, academic standards are not uniform [24]. Referring to the studies of Lo and Cheung [54] and Xu et al. [24], this study measures the disaster experience of residents by the following questions: the severity of the impact of the most severe earthquake disaster on the family in your memory (1 = very not serious, 2 = not serious, 3 = average, 4 = serious, 5 = very serious).

#### 3.2.3. Risk Perception

Disaster risk perception is a concept that describes people’s attitude and intuitive judgment toward disaster risk [22,55]. There are two methods to measure disaster risk perception. The present study mainly followed the psychological measurement method, which presumes that residents’ disaster risk perception is measurable and multi-dimensional. Following the outline by Lennart [56], Slovic [57], Thompson et al. [58], Peng et al. [23,59] and Xu et al. [29,48], this study measured disaster risk perception in two aspects: perceived probability of disaster and perceived severity of disaster. For each dimension, we chose one index to measure it. For example, regarding the measure of residents’ perception of the possibility of disaster, we asked residents how much they agreed with the following statement: there may be a big earthquake near your home in the next 10 years (1 = strongly disagree, 2 = disagree, 3 = average, 4 = agree, 5 = strongly agree).

#### 3.2.4. Control Variables

To improve the power of the model, some factors affecting residents’ disaster risk perception were added as control variables, as performed by Armaş [16], Ho et al. [60], Huang et al. [61], Lazo et al. [62] and Lindell and Perry [20]. These included personal characteristics of study participants (e.g., gender, age, education, etc.), and social and economic characteristics of families (e.g., income, age, children, etc.).

#### 3.2.5. Analytic Strategy

The dependent variable of the present study was residents’ disaster risk perception, which included two indicators: probability and severity of disaster occurrence. Because these two variables are measured by 1–5-point Likert scales, which are regarded as interval variables, the ordinary least square method was used to make estimations of the model. The estimation formula is as follows:

(1) $$Y_{i}=\alpha_{0}+\rho_{1i}\times ME_{i}+\rho_{2i}\times ES_{i}+\rho_{3i}\times Control_{i}+\epsilon_{i}$$

where $Y_{i}$ refers to the probability and severity of disasters respectively; $ME_{i}$ and $ES_{i}$ are core independent variables, which refer to media exposure and the severity of disaster experience; $Control_{i}$ represents the socio-economic characteristics of residents’ individuals and families; $\alpha_{0}$, $\rho_{1i}$, $\rho_{2i}\;$ and $\rho_{3i}$ represent model parameters to be estimated, respectively; $\epsilon_{i}$ refers to residual items.

## 4. Results

### 4.1. Descriptive Statistical Analysis

#### 4.1.1. Media Exposure

Table 2 shows the distribution and frequency of media exposure. Television was the main traditional media source of disaster information for rural households (188 households; 57.49%), with significantly less information coming from newspapers, magazines, and radio (less than 5%). For new media forms, the internet and mobile devices were two important ways for rural households to obtain disaster information. In the research area, all mobile phones can surf the Internet. A total of 137 rural households (41.90%) often obtain disaster information through mobile phones, while 81 households (24.77%) often obtain disaster information through the internet. In contrast, 42.82% and 66.97% of rural households rarely used mobile phones and the internet, respectively, to obtain disaster information.

#### 4.1.2. Disaster Experiences

**Figure 4:**
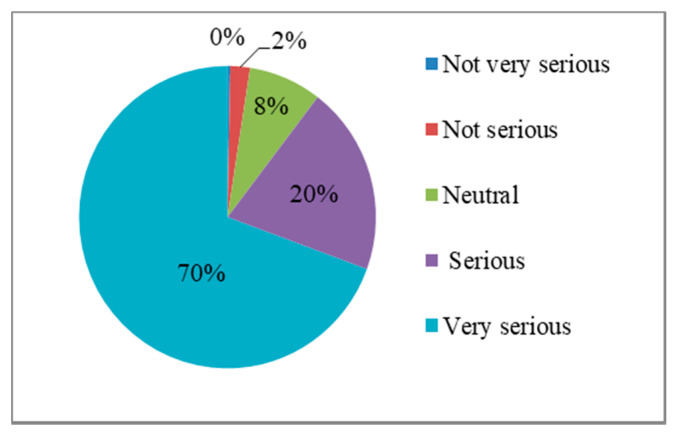
Distribution of disaster experiences frequency.

Figure 4 shows the distribution and frequency of disaster experiences. For the Wenchuan or Lushan earthquakes, 90% of residents thought the disaster experience was serious, while only 2% of residents considered the disaster experience as not serious.

#### 4.1.3. Disaster Risk Perception

**Figure 5:**
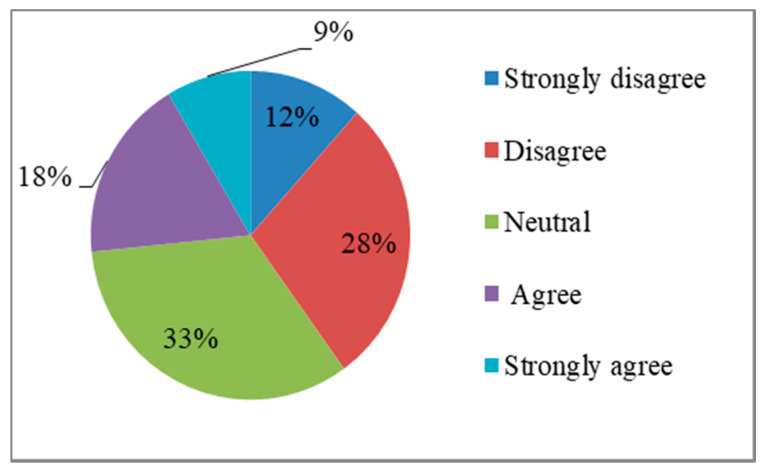
Another major earthquake may occur in the next 10 years.

**Figure 6:**
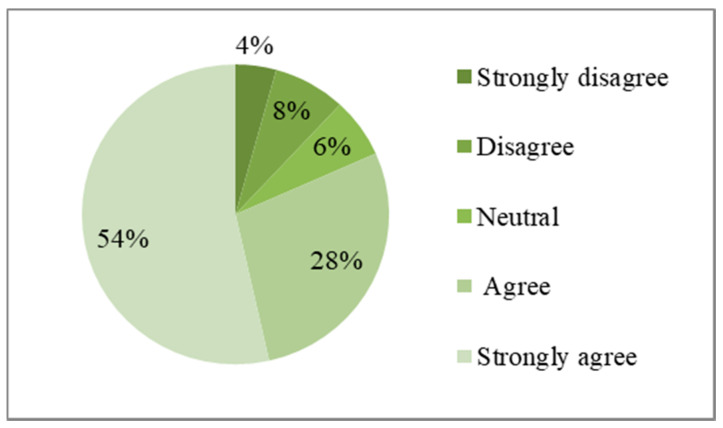
Another major earthquake will seriously affect residents’ lives and safety of property.

Figure 5 shows the distribution of the perceived probability of events. Approximately 27% of residents thought that another major earthquake may occur in the next 10 years, while 40% of residents did not believe this. The remaining 33% of residents were neutral. Figure 6 shows the distribution of perceived severity of events. Approximately 82% of the residents believed that another major earthquake will seriously affect their lives and safety of property, while 12% of the residents did not have these concerns. The remaining 6% of residents held a neutral attitude to this question.

#### 4.1.4. Control Variables

As shown in Table 1, 54% of the respondents were men, the average age was 53.4 years, the average length of education was 6.29 years, and the most common occupation was farming (57%). The average cash income of the respondents’ families was 66,239 Yuan. Almost half of households (48%) included people over 64 years old, while nearly a quarter (24%) included children under 6 years old. Almost half of the households surveyed lived in houses that were constructed of concrete (48%).

### 4.2. Correlation Analysis Among Independent Variables

Table 3 shows the correlation coefficient matrix of the model variables. The correlation coefficient of each independent variable in the model was essentially below 0.5; therefore, there was no significant multicollinearity between the independent variables of the model. With regards to the correlation among media exposure, disaster experience and the perceived prospect ranks of the probability and severity of disasters, the perceived prospect rank of the probability of disasters was significantly negatively correlated with new media exposure and disaster experience, but not with traditional media exposure. The perceived prospect rank of the severity of disasters was significantly and positively correlated with traditional media exposure, while it was not significantly correlated with new media exposure.

Previous studies primarily focused on the correlation between core independent variables and dependent variables and did not control for other variables. The control of other variables, and making correlations between core independent variables and dependent variables, requires further analysis.

Previous studies primarily focused on the correlation between core independent variables and dependent variables and did not control for other variables. The control of other variables, and making correlations between core independent variables and dependent variables, requires further analysis.

### 4.3. Regression Results

Table 4 shows regression analysis results of media exposure, disaster experience and disaster risk perception. Models 1–3 show the regression results of independent variables and the perceived prospect ranks of the probability of disasters. Model 1 is the result when only independent variables concerned were included; Model 2 is the result when the moderating effect of media exposure and experience severity were included, based on Model 1; and Model 3 is the result when the control variables, such as individual and family socio-economic characteristics of respondents, were included, based on Model 2. Models 4–6 display the regression results of independent variables and the perceived severity of disaster, and the settings of each model are essentially the same as those of Models 1–3.

To further explore which media exposures have a greater influence on residents’ perceived prospect ranks of disaster risk perception, media exposure was further divided into traditional and new media exposure forms, based on the analysis in Table 4. An ordinary least square method econometric model was formed to further explore the regression analysis results of traditional and new media exposure, disaster experience, and perceived prospect ranks of risk perception. The settings of each model are the same as those of Models 1–6.

As shown in Table 4 and Table 5, all models passed the overall significance test, indicating that at least one independent variable in the model created by the study had a significant correlation with the dependent variable. Furthermore, according to the R^2^ of the models, all model independent variables explained approximately 6–14% of the variation of the dependent variables.

#### 4.3.1. Correlations between Media Exposure and Perceived Prospect Ranks of Risk Perception

As shown in Table 4, media exposure was negatively correlated with the perceived prospect ranks of the probability and severity of disasters. Thus, the higher the media exposure score, the lower the perceived prospect ranks of the probability and severity of disasters. More precisely, when other conditions remained unchanged, with every one-unit increase in media exposure score, there were 0.343-unit (Model 3) and 0.242-unit (Model 6) reductions in the perceived prospect ranks of the probability and severity of a disaster, respectively.

Table 5 shows that, although traditional and new media exposure forms were negatively correlated with the perceived prospect ranks of the probability and severity of a disaster, traditional media exposure was only significantly negatively correlated with the perceived prospect ranks of the severity of a disaster, while new media exposure was only significantly negatively correlated with the perceived prospect ranks of the probability of a disaster. Specifically, with every one-unit increase in the traditional media exposure score, there was a 0.369-unit reduction in the perceived prospect ranks of the severity of a disaster (Model 14). Likewise, with every one-unit increase in the new media exposure score, there was a 0.137-unit reduction in the perceived prospect ranks of the probability of a disaster (Model 10).

#### 4.3.2. Correlations between Disaster Experiences and Perceived Prospect Ranks of Risk Perception

As shown in Table 4 and Table 5, experience severity was positively and significantly correlated with the perceived prospect ranks of the probability and severity of a disaster. Therefore, the higher the severity score of residents’ disaster experience, the higher their perceived prospect ranks of the probability and severity of a disaster. Specifically, for every one-unit increase in the severity experience score, there were 0.223 (Model 3) and 0.252 (Model 6) unit increases in the perceived prospect ranks of the probability and severity of a disaster, respectively.

#### 4.3.3. Media Exposure Moderates the Relationship between Residents’ Disaster Experience and Perceived Prospect Ranks of Risk Perception

As shown in Table 4, the interaction item (media exposure × experience severity) was significantly negatively correlated with the perceived prospect ranks of severity of a disaster only at the level of 0.1 (Model 5), but was not significantly correlated with the perceived prospect ranks of the probability of a disaster. Therefore, media exposure slightly reduced the severity perception of disaster experience, and thus reduced the perceived prospect ranks of severity of a disaster. Specifically, for every 1 unit increase in the interaction item (media exposure × experience severity), there was a 0.322 unit decrease in the perceived prospect ranks of the severity of a disaster (Model 5).

As shown in Table 5, although there was a negative correlation between traditional media exposure and perceived prospect ranks of the severity of a disaster, the correlation coefficient was not significant. The interaction item (new media exposure × experience severity) was not significantly correlated with the perceived prospect ranks of the probability of a disaster but was significantly negatively correlated with the perceived prospect ranks of the severity of a disaster (Model 13). Therefore, new media exposure influenced the relationship between perceived severity of disaster experience and perceived prospect ranks of the severity of a disaster. Specifically, for every one-unit increase in the interaction item (new media exposure × experience severity), there was a 0.105 unit decrease in the perceived prospect ranks of the severity of a disaster (Model 13).

#### 4.3.4. Correlations between Social and Economic Characteristics of Individuals and Families, and Perceived Prospect Ranks of Risk Perception

As shown in Table 4 and Table 5, aspects of the resident’s age, family income, and having children under 6 years old were significantly correlated with the perceived prospect ranks of the probability of a disaster. Residents who lived longer and had children under the age of six scored higher on the perceived prospect ranks of the probability of a disaster, and the higher the family’s annual cash income, the lower their score for the perceived prospect ranks of the probability of a disaster. In addition, the respondent age was negatively correlated with the perceived prospect ranks of the severity of a disaster, indicating that the older the respondent age, the lower their score of the perceived severity of a disaster. Gender, occupation, ethnicity, occupants aged over 64 years old, and housing material were all not significantly correlated with the perceived prospect ranks of probability and severity of a disaster.

## 5. Discussion

Compared with the existing studies, the marginal contribution of this study is as follows: first, the empirical study analyzes the relationship between media exposure, disaster experience and perceived prospect ranks of risk perception. It is worth mentioning that when exploring the correlation between media exposure and residents’ perceived prospect ranks of disaster risk perception, we further divided media exposure into new media exposure and old media exposure, and respectively explored the correlation between the two and residents’ perceived prospect ranks of disaster risk perception, and obtained some interesting results. Second, the object of this study is the farmers in earthquake disaster threat areas of China. These groups are generally vulnerable due to their resource endowment. However, this group has received relatively little attention in previous studies. In general, the design of the research program, the concern of the research group and the research results can provide some references and inspirations for the formulation and behavioral decision-making of disaster prevention and mitigation policies of the residents in disaster threat areas.

Media exposure is an important factor affecting residents’ perceived prospect ranks of disaster risk perception. However, inconsistent with the research results of Basolo et al. [7], Zhu and Yao [34], Fleming et al. [46] and Hong et al. [10], which found that media exposure could significantly improve residents’ perceived prospect ranks of disaster risk perception, and with research hypothesis H1, the results from the present study showed that media exposure was significantly and negatively correlated with the perceived prospect ranks of the probability and severity of a disaster. This study demonstrated that both traditional and new media forms were important factors affecting residents’ perceived prospect ranks of disaster risk perception. Among them, inconsistent with hypothesis H1a, the study found that the higher the frequency of traditional media used, the lower the score of the perceived prospect ranks of the severity of a disaster. Inconsistent with research hypothesis H1b, the higher the frequency of new media used, the lower the score of the perceived prospect ranks of the probability of a disaster. Possible reasons for this are as follows.

One possibility is the difference in core variables measured. As for the measurement of media exposure, some previous studies only focus on a certain category (for example, Fleming et al. [46] focuses on traditional media newspapers), and some focus on the integration of media (for example, Hong et al. [10] focuses on the media exposure of the combination of traditional media and new media). As for the measurement of perceived prospect ranks of disaster risk perception, most studies do not subdivide it as this study does, but obtain a comprehensive disaster risk perception (for example, Hong et al. [10]’s disaster risk perception is a comprehensive measure of residents’ perception of the severity of various disasters). Different measurement criteria may lead to different research results.

Second, it may be related to the speed and quality of information transmitted by traditional media and new media. The study area of this study is the earthquake disaster threat area in China, and these areas are mostly relatively poor mountainous areas. Driven by economic interests, a large number of young people go out for work, leaving behind relatively old people with relatively few years of education [27,63,64,65,66,67,68,69,70]. In the face of the threat of earthquake disaster, TV is generally the most important traditional media channel for the elderly to obtain disaster information (the results of this study also found that residents get disaster information most frequently from TV). At the same time, with the development of social economy, mobile phones, especially old ones which can only make phone calls and receive information, are widely popular in rural China. Because of the convenience and low cost of receiving information by mobile phone, it often becomes an important channel for grass-roots governments to release official information about disasters and an important carrier for residents to transmit information to each other. Since rural residents generally have a low level of education and are more susceptible to the “rumor” of the epidemic, grass-roots governments generally only promptly report the occurrence of the disaster (such as the magnitude of the aftershocks) and tell residents to pay attention to the safety of life and property when disseminating disaster-related information through new media channels such as mobile phones. Therefore, the new media represented by mobile phones is more to help residents correctly understand the possibility of disaster and improve their perceived prospect ranks of the possibility of disaster. At the same time, the traditional media, represented by TV, generally reports disaster-related information confirmed and approved by the superior government (generally at the level of district, county or above). These messages typically lag behind those received via mobile phone. However, this information can better reflect the actual severity of the disaster (for example, TV news can directly tell residents the specific casualties caused by the earthquake). Therefore, the traditional media, represented by TV, mainly helps residents to correctly understand the seriousness of disasters and improve their perceived prospect ranks of severity of the disasters (For example, residents use TV to learn about casualties and damage caused by disasters).

Third, the sample size of this study is only 327 samples, relatively small, and the results of the model estimate may be discounted. This may also affect the results of this study.

Meanwhile, it is interesting to note that, as shown in Table 2, residents mainly obtain disaster information through TV and mobile phones, which is quite different from other countries. In some other countries, many studies have found that in the face of the impact of disasters, traditional media, such as television and radio, are considered to be the most frequently used channels for residents to receive information [12,71,72,73,74]. Meanwhile, the usefulness of traditional media is higher than that of new media [12], and these results are especially true for remote areas. The possible reasons for this are geographical differences and residents’ usage habits. Limited by geographical location (most of the surveyed areas in this study are mountainous) and usage habits, Chinese rural residents read fewer newspapers and magazines and use fewer radios. The elderly mainly receive disaster information through television news. Although some elderly people do receive disaster information released by the government through mobile phones, younger people are more inclined to use mobile phones and the internet to obtain disaster information. The implication is that the government should consider television, mobile phones, and the internet to publicize or convey disaster-related information in remote hill-top settlements. Moreover, the government should also pay attention to information accuracy and quality, and reduce the spread of false information in disaster-threatened areas. Other means of information dissemination should also be considered to avoid the impact of power failure and network interruption caused by disasters.

Consistent with the research results of H2 and the results of the study by Botzen et al. [50] and Xu et al. [22], the present study found that residents’ disaster experience was significantly and positively correlated with the perceived prospect ranks of probability and severity of a disaster. However, inconsistent with hypotheses H3, H3a and H3b, this study found that media exposure was significantly and negatively correlated with the perceived prospect ranks of the severity of a disaster, but not significantly correlated with the perceived prospect ranks of the probability of a disaster. By subdividing media exposure into traditional and new media exposure, the study found that only new media exposure was significantly negatively correlated with the perceived prospect ranks of the severity of a disaster. Therefore, the type of media platform influences the perceived prospect ranks of the severity of a disaster. The aim is to urge residents to pay attention to information about disaster severity and plan appropriate preparation strategies. Because people are worried about how an earthquake will impact their families and villages, they will pay more attention to the possibility and severity of the disaster. Some studies found the existence of a “negativity bias” phenomenon in behavioral decisions (e.g., [75,76,77,78,79]). In other words, faced with the threat of disaster, residents have a stronger behavioral response to negative news and are more willing to make behavioral decisions in response to negative information. Therefore, before the disaster information is uncertain, residents in earthquake-prone areas (with rich disaster experience) may tend to make a “negative” assessment of the perceived prospect ranks of severity of a disaster. At this time, the rapid transmission of disaster information by new media (mainly mobile phones) (such as clearly telling residents where and how big the earthquake disaster is) can reduce the uncertainty of information and clarify the actual situation of the disaster, thus reducing the awareness of perceived prospect ranks of the severity of a disaster. Therefore, the interaction between new media exposure and the severity of disaster experience was only negatively correlated with the perceived prospect ranks of severity of a disaster.

## 6. Conclusions

Based on the survey data of 327 households in four counties located in the worst-hit areas of the Sichuan earthquake in China, the present study analyzed the characteristics and associations of residents’ media exposure, disaster experience, and perceived prospect ranks of disaster risk perception, and obtained the following conclusions.

(1) Rural households relied predominately on television broadcasts from traditional media, and on mobile phones and internet content from new media, to obtain disaster information. From the residents surveyed, 90% believed that a disaster experience was serious, 82% considered that another major earthquake would seriously affect their lives and property, while approximately 40% of the residents did not believe there would be another major earthquake in the next 10 years.

(2) Media exposure was negatively correlated with the perceived prospect ranks of the probability and severity of a disaster. The higher media exposure, the less likely that residents assign higher ranks to the probability and severity of disasters. Traditional media exposure was only significantly negatively correlated with the perceived prospect ranks of the severity of a disaster, while new media exposure was only significantly negatively correlated with the perceived prospect ranks of probability of a disaster. The experience severity was significantly positively correlated with the perceived prospect ranks of the probability and severity of a disaster. The higher the disaster severity experience, the more likely that residents would assign higher ranks to the probability and severity of disasters.

(3) The moderating effect between media exposure and disaster severity experience was significantly negatively correlated with the perceived prospect ranks of severity of a disaster; therefore, media exposure can slightly reduce the severity of disaster experience, and thus reduce the perceived prospect ranks of the severity of a disaster. The moderating effect between new media exposure and the disaster severity experience was significantly negatively correlated with the perceived prospect ranks of the severity of a disaster, indicating that new media exposure influences the perceived prospect ranks of the severity of a disaster.

This research has some limitations. For example, the study only focused on the association between media exposure, disaster experience, and disaster risk perception in earthquake regions. Whether similar conclusions apply to other types of disasters requires further testing. In addition, this study investigated the impact of the sources of media information and its use frequency on residents’ disaster risk perception, but it did not consider the impact of the quality of media information on residents’ disaster risk perception. Further research should be performed to explore these associations. Additionally, the focus group of this study is the special group of farmers in the earthquake disaster threat areas of China. Due to the differences in resource endowment and cultural background, whether the research results are applicable to the urban residents threatened by the disaster needs to be further verified.

## Figures and Tables

**Table 1:** Definition and descriptive statistics of the variables in the model.

Category	Variable	Definition and Measure	Mean	Median	SD ^e^
Risk perception	Possibility	There may be a big earthquake near your home in the next 10 years ^a^	2.83	3.00	1.12
	Severity	An earthquake in the future will have a serious impact on villages and rural households ^b^	4.19	3.00	1.12
Media exposure	Traditional channel	How often do you read newspapers? ^c^	1.07	3.00	0.40
	Traditional channel	How often do you read a magazine? ^c^	1.06	3.00	0.44
	Traditional channel	How often do you listen to the radio? ^c^	1.09	3.00	0.51
	Traditional channel	How often do you watch TV? ^c^	3.67	3.00	1.24
	New channel	How often do you use your mobile phone? ^c^	2.12	3.00	1.58
	New channel	How often do you use the Internet? ^c^	2.90	3.00	1.66
Disaster Experience	Experience severity	The severity of residents’ disaster experience ^b^	4.56	3.00	0.76
Individual characteristics	Gender	Responder gender (0 = male, 1 = female)	0.46	0.00	0.50
	Age	Responder age (year)	53.44	5.300	13.40
	Education	Years of education (year)	6.29	6.00	3.70
	Residence	Length of residence of responder (year)	41.71	45.00	19.78
	Nationality	Responder nationality (0 = other, 1 = Han)	0.82	0.00	0.39
	Occupation	Responder occupation (0 = other, 1 = Farmer)	0.57	1.00	0.50
Household characteristics	Income	Total annual cash income of rural households (Yuan ^d^)	66,238.94	46,200.00	72,237.87
	Old	Whether the resident family comprises individuals over 64 years of age (0 = no, 1 = yes)	0.48	0.00	0.50
	Child	Whether the resident family has a child below 6 years of age (0 = no, 1 = yes)	0.24	0.00	0.43
	House	Whether the house is a concrete structure (0 = no, 1 = yes)	0.48	0.00	0.50

Note: ^a^ 1 = strongly disagree, 2 = disagree, 3 = average, 4 = agree, 5 = strongly agree; ^b^ 1 = not very serious, 2 = not serious, 3 = general, 4 = serious, 5 = very serious; ^c^ 1 = never, 2 = rarely, 3 = average, 4 = often, 5 = very often; ^d^ 1 USD = 6.88 Yuan (at the time of the study); ^e^ SD = standard deviation.

**Table 2:** Distribution of media exposure frequency.

Media Exposure		Never	Rarely	Average	Often	Very Often
**Traditional Channel**	Newspapers	314(96.02%)	7(2.14%)	3(0.92%)	2(0.61%)	1(0.31%)
	Magazine	319(97.55%)	1(0.31%)	4(1.22%)	0(0.00%)	3(0.92%)
	Radio	316(96.64%)	3(0.92%)	2(0.61%)	3(0.92%)	3(0.92%)
	TV	25(7.65%)	29(8.87%)	85(25.99%)	77(23.55%)	111(33.94%)
**New Channel**	Mobile phone	118(36.09%)	22(6.73%)	50(15.29%)	48(14.68%)	89(27.22%)
	Internet	203(62.08%)	16(4.89%)	27(8.26%)	29(8.87%)	52(15.90%)

**Table 3:** Correlation coefficient matrix of model variables.

Variable	1	2	3	4	5	6	7	8	9	10	11	12	13	14	15
1	1														
2	0.199 ***	1													
3	−0.085	−0.158 ***	1												
4	−0.203 ***	−0.068	0.119 **	1											
5	0.154 ***	0.188 ***	−0.068	−0.007	1										
6	−0.077	0.062	−0.084	0.017	0	1									
7	0.104 *	−0.047	−0.058	−0.484 ***	−0.017	−0.212 ***	1								
8	−0.140 **	−0.109 **	0.136 **	0.455 ***	−0.044	−0.136 **	−0.496 ***	1							
9	−0.105 *	−0.063	0.02	0.041	−0.04	−0.06	−0.002	0.177 ***	1						
10	0.045	−0.004	−0.021	−0.295 ***	0.026	0.102 *	0.271 ***	−0.371 ***	−0.05	1					
11	0.164 ***	0.017	−0.05	−0.261 ***	−0.031	−0.268 ***	0.517 ***	−0.343 ***	−0.036	0.161 ***	1				
12	−0.027	0.028	−0.017	−0.241 ***	−0.024	−0.135 **	0.272 ***	−0.185 ***	0.072	0.076	0.231 ***	1			
13	0.109 **	0.032	0.042	0.111 **	0.005	0.0790	−0.178 ***	0.176 ***	0.079	−0.078	−0.148 ***	−0.074	1		
14	−0.094 *	−0.108 *	0.178 ***	0.233 ***	0.005	0.0430	−0.132 **	0.261 ***	0.117 **	−0.152 ***	−0.231 ***	−0.158 ***	0.087	1	
15	−0.142 **	−0.156 ***	0.130 **	0.241 ***	0.100 *	−0.0580	−0.143 ***	0.245 ***	0.056	−0.237 ***	−0.023	−0.034	0.116 **	0.210 ***	1

Note: *** *p* < 0.01, ** *p* < 0.05, * *p* < 0.1; 1 = possibility, 2 = severity, 3 = traditional channel, 4 = new channel, 5 = experience severity, 6 = gender, 7 = age, 8 = education, 9 = nationality, 10 = occupation, 11 = residence, 12 = old, 13 = child, 14 = house, 15 = income.

**Table 4:** Regression analysis results of media exposure, disaster experience and perceived prospect ranks of risk perception.

Variables	Possibility			Severity		
	Model 1	Model 2	Model 3	Model 4	Model 5	Model 6
Media exposure	−0.402 ***	−0.401 ***	−0.343 ***	−0.235 **	−0.233 **	−0.242 *
	(0.108)	(0.107)	(0.123)	(0.114)	(0.112)	(0.146)
Experience severity	0.217 ***	0.205 ***	0.223 ***	0.273 ***	0.255 ***	0.252 ***
	(0.076)	(0.075)	(0.074)	(0.098)	(0.094)	(0.096)
Media exposure * Experience severity		−0.212	−0.163		−0.322 *	−0.277
		(0.147)	(0.142)		(0.176)	(0.182)
Gender			−0.200			0.047
			(0.137)			(0.126)
Age			−0.004			−0.013 **
			(0.006)			(0.006)
Education			−0.017			−0.030
			(0.023)			(0.023)
Nationality			−0.257			−0.099
			(0.159)			(0.159)
Occupation			−0.081			−0.117
			(0.133)			(0.133)
Residence			0.008 **			0.002
			(0.004)			(0.004)
Ln(income)			−0.117 *			−0.010
			(0.069)			(0.083)
Old			−0.197			0.081
			(0.122)			(0.126)
Child			0.456 ***			0.115
			(0.144)			(0.139)
House			0.011			−0.137
			(0.132)			(0.131)
Constant	2.645 ***	2.694 ***	4.045 ***	3.409 ***	3.483 ***	4.535 ***
	(0.389)	(0.392)	(0.841)	(0.557)	(0.539)	(1.044)
F	10.030 ***	8.185 ***	4.338 ***	7.831 ***	5.752 ***	2.096 **
R^2^	0.066	0.071	0.139	0.050	0.063	0.092
Observations	327	327	327	327	327	327

Note: Robust standard errors in parentheses; *** *p* < 0.01, ** *p* < 0.05, * *p* < 0.1.

**Table 5:** Regression analysis results of traditional and new media exposure, disaster experience and perceived prospect ranks of risk perception.

Variables	Possibility				Severity			
	Model 7	Model 8	Model 9	Model 10	Model 11	Model 12	Model 13	Model 14
Traditional channel	−0.160	−0.156	−0.142	−0.111	−0.434 ***	−0.428 **	−0.412 **	−0.349 *
	(0.172)	(0.170)	(0.171)	(0.170)	(0.164)	(0.171)	(0.164)	(0.185)
New channel	−0.149 ***	−0.149 ***	−0.155 ***	−0.137 ***	−0.039	−0.038	−0.046	−0.049
	(0.042)	(0.042)	(0.042)	(0.048)	(0.044)	(0.044)	(0.044)	(0.055)
Experience severity	0.220 ***	0.222 ***	0.200 **	0.220 ***	0.265 ***	0.268 ***	0.240 **	0.238 **
	(0.076)	(0.078)	(0.078)	(0.078)	(0.098)	(0.099)	(0.093)	(0.096)
X1		−0.052		−0.009		−0.080		−0.031
		(0.217)		(0.203)		(0.324)		(0.326)
X2			−0.083	−0.068			−0.105 *	−0.098
			(0.055)	(0.052)			(0.060)	(0.061)
Gender				−0.189				0.043
				(0.138)				(0.125)
Age				−0.005				−0.012 *
				(0.006)				(0.007)
Education				−0.016				−0.031
				(0.023)				(0.023)
Nationality				−0.256				−0.100
				(0.160)				(0.159)
Occupation				−0.090				−0.097
				(0.136)				(0.133)
Residence				0.008 **				0.002
				(0.004)				(0.004)
Ln(income)				−0.117 *				−0.009
				(0.070)				(0.084)
Old				−0.199				0.095
				(0.122)				(0.127)
Child				0.454 ***				0.119
				(0.144)				(0.138)
House				0.002				−0.130
				(0.133)				(0.133)
Constant	2.482 ***	2.463 ***	2.555 ***	3.949 ***	3.827 ***	3.797 ***	3.919 ***	4.774 ***
	(0.468)	(0.469)	(0.467)	(0.863)	(0.584)	(0.614)	(0.564)	(1.075)
F	6.729 ***	5.033 ***	6.536 ***	3.831 ***	6.397 ***	4.918 ***	4.930 ***	2.224 ***
R-squared	0.067	0.067	0.074	0.142	0.059	0.059	0.070	0.097
Observations	327	327	327	327	327	327	327	327

Note: Robust standard errors in parentheses; *** *p* < 0.01, ** *p* < 0.05, * *p* < 0.1; X1 and X2 refer to traditional channel × experience severity and new channel × experience severity, respectively.
